# Assessing Elevated Blood Glucose Levels Through Blood Glucose Evaluation and Monitoring Using Machine Learning and Wearable Photoplethysmography Sensors: Algorithm Development and Validation

**DOI:** 10.2196/48340

**Published:** 2023-10-27

**Authors:** Bohan Shi, Satvinder Singh Dhaliwal, Marcus Soo, Cheri Chan, Jocelin Wong, Natalie W C Lam, Entong Zhou, Vivien Paitimusa, Kum Yin Loke, Joel Chin, Mei Tuan Chua, Kathy Chiew Suan Liaw, Amos W H Lim, Fadil Fatin Insyirah, Shih-Cheng Yen, Arthur Tay, Seng Bin Ang

**Affiliations:** 1 Actxa Pte Ltd Singapore Singapore; 2 Activate Interactive Pte Ltd Singapore Singapore; 3 Curtin Health Innovation Research Institute Curtin University Perth Australia; 4 Faculty of Health Sciences Curtin University Perth Australia; 5 Duke-NUS Graduate Medical School National University of Singapore Singapore Singapore; 6 KK Women’s and Children’s Hospital Singapore Singapore; 7 Innovation and Design Programme Faculty of Engineering National University of Singapore Singapore Singapore; 8 Department of Electrical and Computer Engineering National University of Singapore Singapore Singapore; 9 Family Medicine Academic Clinical Program Duke-NUS Medical School Singapore Singapore; 10 Menopause Unit KK Women’s and Children’s Hospital Singapore Singapore

**Keywords:** diabetes mellitus, explainable artificial intelligence, feature engineering, machine learning, photoplethysmography, wearable sensor

## Abstract

**Background:**

Diabetes mellitus is the most challenging and fastest-growing global public health concern. Approximately 10.5% of the global adult population is affected by diabetes, and almost half of them are undiagnosed. The growing at-risk population exacerbates the shortage of health resources, with an estimated 10.6% and 6.2% of adults worldwide having impaired glucose tolerance and impaired fasting glycemia, respectively. All current diabetes screening methods are invasive and opportunistic and must be conducted in a hospital or laboratory by trained professionals. At-risk participants might remain undetected for years and miss the precious time window for early intervention to prevent or delay the onset of diabetes and its complications.

**Objective:**

We aimed to develop an artificial intelligence solution to recognize elevated blood glucose levels (≥7.8 mmol/L) noninvasively and evaluate diabetic risk based on repeated measurements.

**Methods:**

This study was conducted at KK Women’s and Children’s Hospital in Singapore, and 500 participants were recruited (mean age 38.73, SD 10.61 years; mean BMI 24.4, SD 5.1 kg/m^2^). The blood glucose levels for most participants were measured before and after consuming 75 g of sugary drinks using both a conventional glucometer (Accu-Chek Performa) and a wrist-worn wearable. The results obtained from the glucometer were used as ground-truth measurements. We performed extensive feature engineering on photoplethysmography (PPG) sensor data and identified features that were sensitive to glucose changes. These selected features were further analyzed using an explainable artificial intelligence approach to understand their contribution to our predictions.

**Results:**

Multiple machine learning models were trained and assessed with 10-fold cross-validation, using participant demographic data and critical features extracted from PPG measurements as predictors. A support vector machine with a radial basis function kernel had the best detection performance, with an average accuracy of 84.7%, a sensitivity of 81.05%, a specificity of 88.3%, a precision of 87.51%, a geometric mean of 84.54%, and *F* score of 84.03%.

**Conclusions:**

Our findings suggest that PPG measurements can be used to identify participants with elevated blood glucose measurements and assist in the screening of participants for diabetes risk.

## Introduction

Diabetes mellitus (DM) is a chronic and heterogeneous metabolic disorder characterized by the presence of hyperglycemia due to deterioration of insulin secretion, defective insulin action, or both [1,2]. There are 3 main types of DM: type 1 DM (T1DM), type 2 DM (T2DM), and gestational diabetes. T2DM is the most prevalent type of diabetes, affecting over 95% of people with diabetes worldwide [3,4].

The prevalence of DM has been proliferating in recent decades, and it is now the most prominent and fastest-growing global public health challenge [5,6]. Uncontrolled diabetes is associated with an increased risk of complications such as cardiovascular disease, kidney failure, vision loss, nerve damage, and overall mortality [7-9]. On the basis of the latest diabetes prevalence estimate, 10.5% of the global adult population is affected by diabetes, and almost half of them are undiagnosed [10]. The growing at-risk population has further strained scarce health resources. Globally, approximately 10.6% of adults have impaired glucose tolerance (IGT) and 6.2% have impaired fasting glycemia (IFG) [4]. IGT and IFG are reversible transitional conditions between normality and diabetes. These conditions, also known as prediabetes, are characterized by elevated blood glucose levels that are not high enough to be classified as diabetes. However, individuals with IGT or IFG are at increased risk of developing cardiovascular disease, coronary heart disease, stroke, and mortality [11]. One of the challenges with IGT and IFG is that they often do not have any obvious symptoms, which means that they can go undetected and undiagnosed for years. Moreover, a follow-up study conducted in Singapore reported that one-third of these individuals with prediabetes would likely develop T2DM within 8 years without lifestyle changes [12]. A similar study with data from the United Kingdom has also reported that a substantial proportion of individuals with prediabetes could progress to T2DM within 5 years [13]. Therefore, predicting the risk of diabetes in the asymptomatic population is a significant health challenge that must be addressed. Early recognition of prediabetes and undiagnosed T2DM will result in a better health outcome or a more favorable long-term prognosis [14].

Currently, the diagnosis of diabetes and prediabetes is well established. T2DM and prediabetes can be detected using one of four methods: (1) the fasting plasma glucose value, (2) the 2-hour plasma glucose value during a 75 g oral glucose tolerance test, (3) hemoglobin A_1c_, and (4) a random plasma glucose test [3]. All these diagnostic screening methods are invasive and opportunistic in nature and must be conducted in a hospital or laboratory by trained professionals. A confirmed diagnosis usually requires repeated testing. As all the tests are single-time point screenings, adults aged >35 years are recommended to undergo regular screening every 3 years. Nevertheless, at-risk individuals hardly comply with this recommendation, especially in developing countries, owing to the cost of diagnostic tests and the scarcity of medical resources [15,16].

Unlike T1DM and gestational diabetes, the development of T2DM and its complications is preventable or controllable. A considerable number of studies have shown that lifestyle and behavioral interventions help patients with diabetes achieve adequate glycemic control [17,18]. Recent evidence also suggests that early lifestyle adjustment will help participants with prediabetes return to normoglycemia and reduce the risk of developing T2DM [19-21]. Frequent diabetes screening identifies individuals with a high risk of T2DM 2.2 years earlier [22], creating a precious time frame and opportunity for taking an early intervention to prevent or delay the onset of diabetes and its complications and improve overall clinical outcomes.

For established individuals with diabetes, constant monitoring of their blood glucose concentration is crucial so that appropriate insulin dosage can be administered in a timely manner to avoid acute and chronic complications and delay disease progression. Conventional blood glucose measurement requires patients to prick their fingers several times a day, which causes the development of massive scarring and loss of sensation at the fingertips over the year [23]. This measurement method is invasive, inconvenient, and expensive, which are the main barriers to the effective self-management of diabetes in the older adult group [24,25]. To improve diabetes outcomes and assist patients in self-managing the disease, continuous glucose monitoring devices have entered the market and are made available for some patients with diabetes. However, most continuous glucose monitoring sensors currently available are still invasive, which measures glucose concentration in the subcutis using an electrochemical needle sensor [26]. Users need to replace the sensor frequently and purchase different components of the system regularly, which will cost from US $2500 to US $6000 per year [27,28].

In recent years, the advancement and use of wearable technologies and artificial intelligence (AI) have gradually changed our daily lives, as many people use wrist-worn wearables daily for fitness and health monitoring [29]. Most consumer wearables have incorporated green light reflection photoplethysmography (PPG) sensors into their products. Wearable technology has the potential to greatly expand the impact of public health initiatives by using a proactive approach to identify abnormal physiological signals, assessing disease risk factors, and helping patients manage chronic conditions and recovery [30-33].

In 2011, Monte-Moreno [34] demonstrated the use of PPG data collected using a pulse oximeter to estimate blood glucose levels. By analyzing the PPG waveform, features such as the respiration frequency, heart rate variability (HRV), and other physiological parameters can be extracted. They are then fed into a random forest model, yielding a prediction accuracy of 87.7% based on the Clark error grid. Rodin et al [35] validated a wearable biosensor developed by Zilberstein et al [36] as an indirect measure of glucometry. The biosensor comprises a PPG sensor and an optically sensitive backglass panel that changes its optochemical characteristics according to the concentrations of specific sweat metabolites. In total, 200 adult participants were recruited, and each participant wore a smartwatch to extract PPG data, while blood samples were collected from the antecubital vein concurrently. The estimation of the blood glucose level was derived using a proprietary algorithm developed by SpectroPhon and compared against a glucose lactate analyzer (YSI 2300). The proposed biosensor was able to detect anteprandial glucose with a mean absolute percentage error of 7.4% and a normalized root mean squared error of 11.56%, while postprandial glucose measurements yielded 7.54% mean absolute percentage error and 9.79% normalized root mean squared error. Zhang et al [37] used a smartphone, taking a video of the index finger covering the flash, to capture the fluctuation in the light absorption associated with the change in blood volume. The resulting red, green, and blue image was then transformed into PPG data. The Gaussian fitting method was applied to model the PPG waveform components, from which 28 time-domain and frequency-domain features were extracted. A support vector machine (SVM) with a Gaussian kernel was trained with data from 80 participants to classify the user’s glucose level as normal, borderline, or warning, with an accuracy of 81.49%, 79.85% sensitivity, 83.19% specificity, and 80.2% *F* score. The study was conducted in a highly controlled environment with limited participants, so the generalizability of these results is subject to certain limitations.

Conventional blood glucose monitoring technologies often require invasive measures such as finger pricking or the use of skin sensors and patches. These methods can be uncomfortable and inconvenient for users and can also be financially burdensome. To address these issues, we propose a novel solution called blood glucose evaluation and monitoring (BGEM) that leverages the latest advancements in signal processing, wearable technology, and AI to detect elevated blood glucose levels and evaluate the risk of developing diabetes. With BGEM, users only need to measure their PPG data using a consumer-grade wrist-worn wearable device. The AI model will then compute relevant digital biomarkers and evaluate the risk of prediabetes or T2DM by recognizing elevated blood glucose levels (≥7.8 mmol/L). This solution allows for frequent blood glucose testing without the discomfort and inconvenience of current technologies.

## Methods

### PPG Sensor

**Figure 1:**
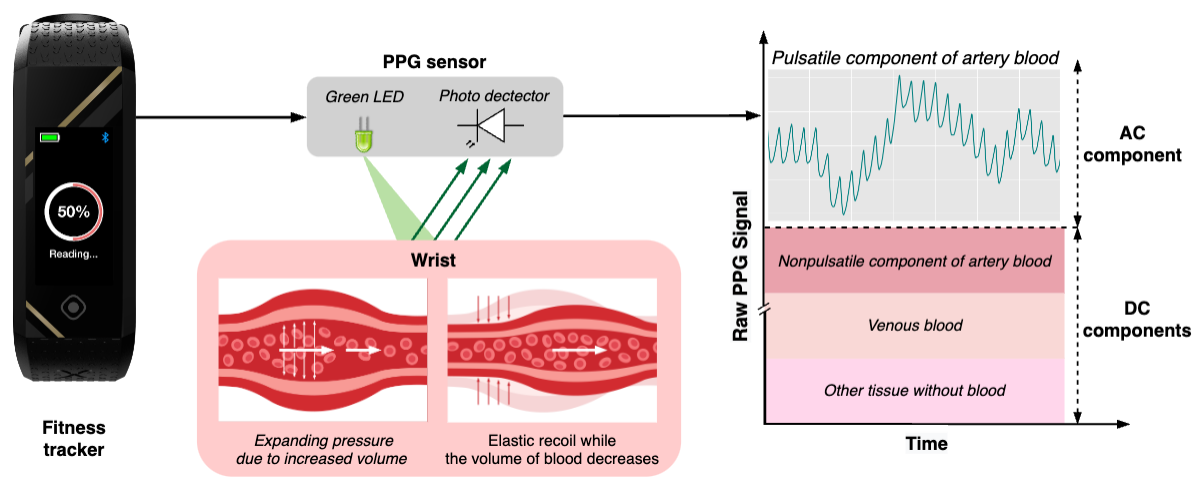
Illustration of the working principle of a photoplethysmography (PPG) sensor. Changes in blood flow represent different phases within the cardiac cycle. During the diastolic phase, blood volume, arterial diameter, and hemoglobin concentration in the measurement site are minimized, leading to minimum absorption of light by blood and, consequently, an increase in light intensity detected by the sensor system. The reverse is valid for the systolic phase, where a decrease in light intensity is detected instead. AC: alternating current; DC: direct current.

PPG is a low-cost, noninvasive technique that measures the volumetric fluctuation in arterial blood flow [38]. The human wrist is one of the sites for measuring the PPG signal because it has a rich arterial source and an excellent sensor placement with minimal interference to one’s daily activities. The PPG signal comprises superimposed pulsatile alternating current components and direct current voltage components. A PPG signal is obtained by illuminating the light emitting device on the skin surface and measuring the variations in light absorption or reflection that reflect the pulsatile flow patterns, as shown in Figure 1.

The pulsatile alternating current component corresponds to the cardiac cycle, characterizing that the wrist’s blood vessels expand and contract with each heartbeat, whereas the direct current component reflects constant light absorption by venous and arterial blood, as well as other tissues [39]. The PPG signal can detect vascular changes associated with diabetes and contains substantial valuable information from HRV, which is significantly associated with diabetes [40]. Hence, it will be used in this study to extract valuable and meaningful features to identify an individual’s glucose status (elevated or normal).

### Ethical Considerations

Before commencing the study, ethical clearance was obtained from the SingHealth Centralised Institutional Review Board of Singapore (2020/2968) on March 21, 2021. All methods were performed in accordance with Singapore’s clinical guidelines and regulations. Informed consent was obtained from all the trial participants or their legal guardians. The clinical trial was registered on ClinicalTrials.gov (NCT05504096) on August 17, 2022.

### Study Protocol

In total, 500 participants were recruited from KK Women’s and Children’s Hospital in Singapore. Participants’ demographics are summarized in Table 1. For most participants, the blood glucose levels were measured before and after consumption of 75 g of a sugary drink using both the conventional glucometer (Accu-Chek Performa) and the wrist-worn wearable device. Participants who were excluded for the second measurement had high blood glucose measurements ≥11.1 mmol/L on their first measurement and hence were not administered the sugary drink measuring 75 g.

**Figure 2:**
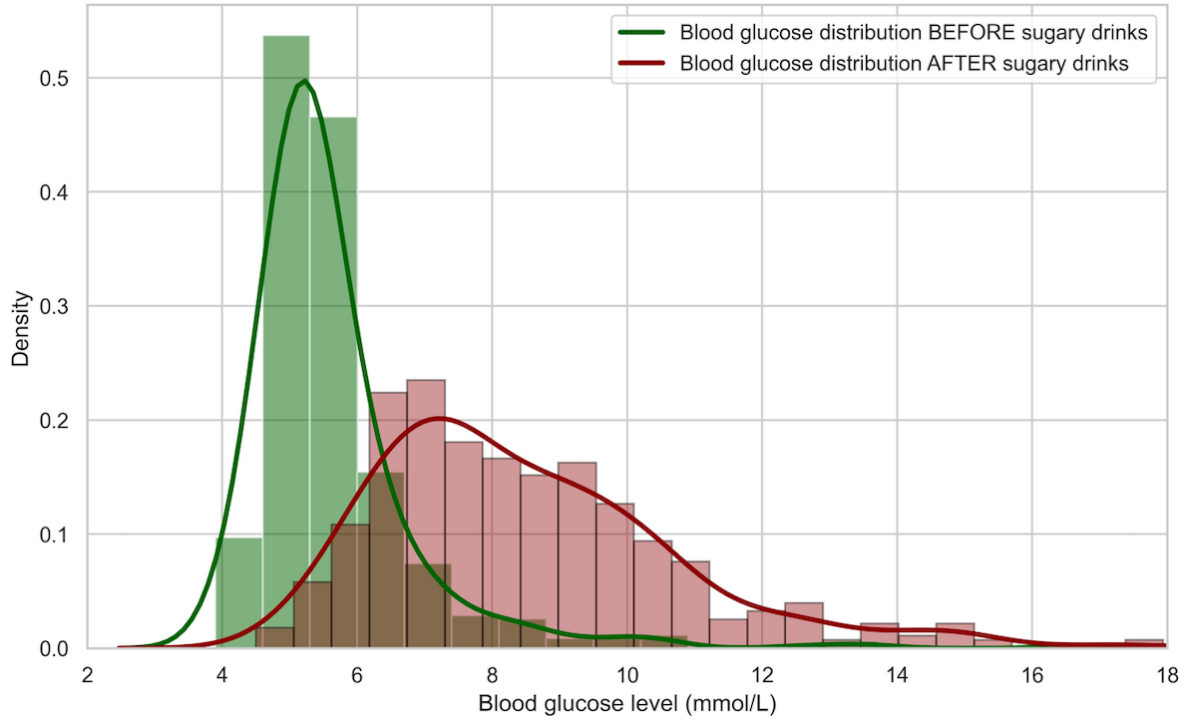
The distribution of ground-truth blood glucose levels before and after sugary drinks (*P*<.001).

After consuming the sugary drink, 55.1% (266/483) of the participants had high blood glucose (≥7.8 mmol/L). The distribution of blood glucose levels before and after consuming the sugary drink is shown in Figure 2. A statistically significant difference was observed between the 2 distributions (*P*<.001).

**Table 1:** Description of participants (N=500).

Characteristics				Values
**Demographic data**				
	Age (years), mean (SD); range			38.73 (10.61); 21-81
	BMI (kg/m^2^), mean (SD); range			24.4 (5.1); 16.3-71.1
	**Gender, n (%)**			
		Men	51 (10.2)	
		Women	449 (89.8)	
**Diabetes profile**				
	**Family history of diabetes, n (%)**			
		Yes	157 (31.4)	
		No	343 (68.6)	
	**Prediabetes, n (%)**			
		Yes	17 (3.4)	
		No	483 (96.6)	
	**Diabetes, n (%)**			
		Yes	8 (1.6)	
		No	492 (98.4)	
	**Gestational diabetes, n (%)**			
		Yes	21 (4.2)	
		No	428 (85.6)	
		N/A^a^	51 (10.2)	

^a^N/A: not applicable.

### Study Device

The Actxa Spark+ Series 2, a low-cost and commercially available wrist-worn wearable device, was used in this project. This multifunctional device, built for everyday activities, fitness, and preventive health monitoring, provided an adequate PPG signal quality at 50 Hz. The wearable device is equipped with advanced PPG technology that enables accurate and reliable measurement of heart rate (HR) and other physiological parameters. This is similar to the devices used in Singapore’s nationwide health care campaigns, such as the National Steps Challenge. It is also worth noting that our proposed solution is device agnostic and can be easily integrated into other wearables with PPG capabilities, allowing for a scalable and cost-effective assessment of risk-based populations, including high-risk participants, participants with undiagnosed diabetes, and patients in need of primary prevention interventions.

### Before Processing

The raw PPG signal was collected using both wrist-worn wearables in 16-bit binary format. We first performed a digital-to-analog conversion using the following formula:



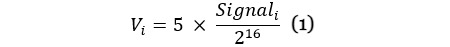



Liang et al [41] suggested that a fourth-order Chebyshev II filter provides an optimal processing performance for short PPG signals. Hence, we adopted the recommended filter design to remove low-frequency drift and high-frequency noise using a band-pass Chebyshev II filter. The proposed band-pass filter has a lower cut-off frequency of 0.3 Hz and an upper cut-off frequency of 4 Hz.

**Figure 3:**
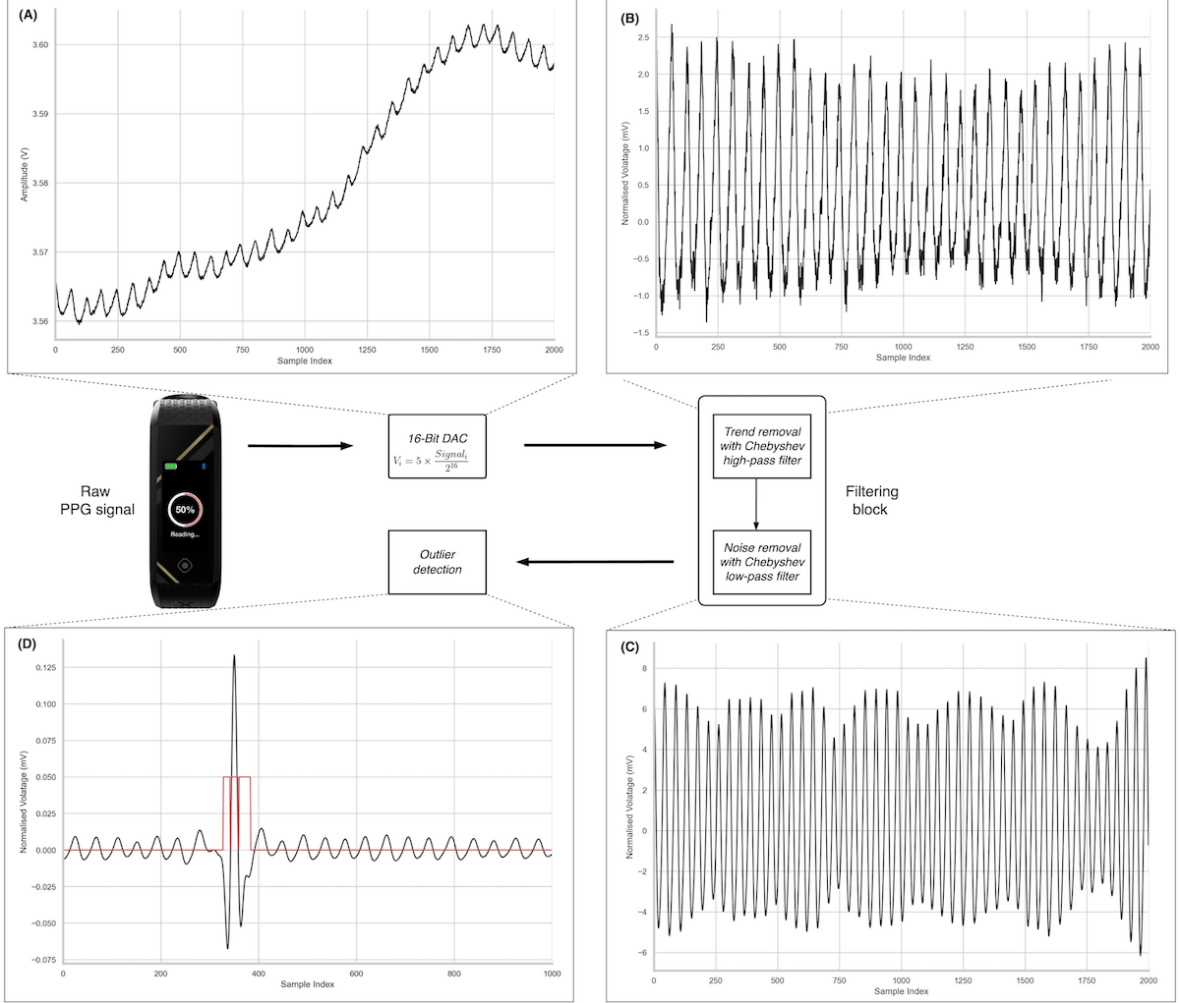
Data preprocessing workflow. (A) Raw photoplethysmography (PPG) signal, (B) removal of the signal’s moving trend using a Chebyshev high-pass filter, (C) use of a Chebyshev low-pass filter to eliminate high-frequency noise, and (D) final step involves outlier identification from the filtered PPG signal. DAC: digital-to-analog conversion.

The filtered PPG signals still contain various forms of outliers, such as peaks with abnormally high amplitudes or distortions in the oscillating waveform, which can be caused by movement from the upper extremity or improper contact between the sensor and skin. Features derived from signals that possess outliers may not be accurate, so a *z* scores outlier detection with a cut-off value of 3 SDs of the mean was applied. The identified outliers or regions of outliers were replaced with a reasonable estimate via a nearest neighbor interpolation for the HRV feature extraction. Because PPG signals do not change drastically in such a short duration, this method is determined to be an appropriate approach to the problem. Furthermore, the number of outliers was minimal in our data set, and hence should not have affected the features that we generated later. The data preprocessing steps are illustrated in Figure 3.

### Feature Extraction

#### Overview

The preprocessed data were suitable for generating reliable features, and a total of 248 features were generated. These features can be classified into seven categories: (1) HRV features, which encompass time domain, frequency domain, and nonlinear HRV features; (2) waveform features; (3) HR features; (4) energy measure features; (5) complexity measure features; (6) continuous wavelet transform (CWT) features; and (7) patient demographics. The complete set of features analyzed in this study is summarized in Multimedia Appendix 1. However, these 248 feature candidates are not all relevant to the change in glucose level, and redundant features might cause prediction performance deterioration. The details of the feature-engineering and feature-selection process are discussed in the “Feature Selection” section.

#### HRV Features

HRV is the variation in time intervals between consecutive heartbeats and is widely used as a noninvasive physiological biomarker of the autonomic nervous system response [42-44]. HRV provides a proxy to measure sympathetic nervous system (SNS) and parasympathetic nervous system (PNS) activity, which reflects the ability to respond to and recover from abrupt physical, psychological, and environmental changes [44-46]. As HR estimated at any given time represents the net effect of the neural output of the PNS, which slows HR, and SNS, which accelerates HR, HRV also detects imbalance in the autonomic nervous system resulting from over- or understimulation of SNS and PNS. Therefore, the fluctuation in HRV values provide useful insights into many clinical applications, such as mental stress, exercise and rehabilitation, cardiovascular fitness, pathological state, progression of chronic disease, and even predicting the onset of diseases [47-51]. Depending on the application, HRV features are usually extracted from an ultra–short-term (*<*5 min), short-term (approximately 5 min), or whole-day 24-hour time frame [52]. Most HRV features can be grouped under time-domain, frequency-domain, or nonlinear categories. In this project, most of the widely used HRV features were included in our analysis and were extracted using a 5-minute time frame. These HRV features are briefly explained in Multimedia Appendix 1 using the feature indices (F1-F71).

#### HR Features

Prior studies have noted the influence of impaired blood glucose on HR, especially resting HR [53,54]. Hence, HR was extracted by finding the number of peaks for every 10 seconds of the filtered PPG signal. The statistical features of the HR were then calculated and used as part of the feature inputs (F72-F81).

#### Wavelet Analysis

A considerable number of studies have applied wavelet transformation to analyze HRV data associated with a wide variety of health care applications. Earlier research has used features derived from CWT to predict blood glucose levels [55]. In this project, we applied CWT to the PPG signal using the Mexican Hat mother wavelet. The mean, SD, and maximum value of the resulting CWT matrix were included in the feature vector (F82-F84).

#### Waveform Features

Previous studies have reported that the characteristics of the PPG waveform extracted from healthy participants and participants with diabetes exhibited statistical differences [37,56]. Nirala et al [56] also suggested that the first and second eigenvalues derived from the first derivative of the PPG signal are the top features for identifying T2DM. In addition, several studies have revealed a functional relationship between the PPG signal and blood glucose levels [34,57]. Similarly, respiratory information can also be extracted from the PPG waveform [33,58]. However, PPG waveforms derived from signals using a wrist-worn PPG sensor often have a nondetectable diastolic peak and a dicrotic notch, unlike the signals collected using fingertip PPG.

**Figure 4:**
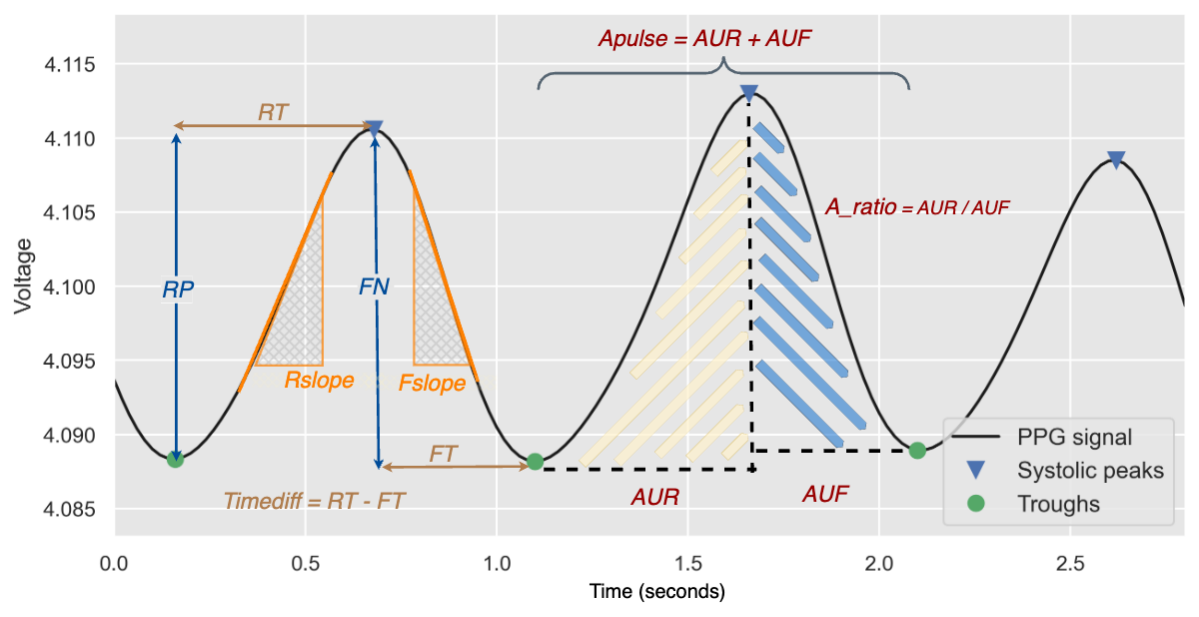
Definition of the photoplethysmography (PPG) waveform features. AUF: area under the falling edge; Apulse: area under a PPG wave; AUR: area under the rising edge; FN: magnitude of falling edge; Fslope: slope of falling edge; FT: fall time; RP: magnitude of rising edge; Rslope: slope of rising edge; RT: rise time.

Waveform features (F85-F196) derived from the PPG waveform were included in the feature set, and the definition of the waveform features is illustrated in Figure 4.

#### Energy Measures

Several studies have used the energy features extracted from PPG signals to estimate blood glucose [34,59,60]. The Kaiser-Teager energy (KTE) operator and logarithmic energy are 2 commonly used methods to analyze the energy profile. These features were computed from a 5-second sliding window, as it ensures that the PPG signals within each window would be long enough to contain several heartbeats but short enough such that the wave amplitude changes are negligible.

The KTE operator is a well-known method for providing a time-frequency analysis of the instantaneous energy of the PPG signal from the amplitude and frequency. Using the implementation strategy explained by Monte-Moreno [34], we computed the energy profile of the PPG signal at each sliding window frame, and the KTE operator for the n-th frame was computed using the following equation:

KTE_n_(i) = x_frame_(i)^2^ – x_frame_(i + 1) * x_frame_(i – 1), which holds for i = 2,3,...,(L_frame_ – 1)
**(2)**

Where *x_frame_* is the filtered PPG signal within each sliding window frame.

The statistical metrics were computed for each frame, and the average of the metrics for the nth frame was then calculated and represented as F197 to F206.

To estimate the respiration rate from the PPG signal, we used the logarithmic energy value calculated at the frame level using the following equation:



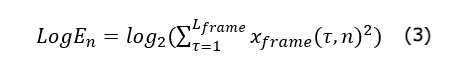



Where *x_frame_* is the filtered PPG signal within each sliding window frame.

The autoregressive model coefficients of order 7 were estimated using the Yule-Walker method, and the Python function *aryule* was used for this purpose. In addition, other statistical parameters were also computed (F207-F223).

#### Complexity Measures

Sample entropy (SampEn, F224) measures the unpredictability of physiological signals and is commonly used in HRV analysis [61]. The lower the SampEn, the more regular the signal.

SampEn can be defined after calculating the template vector ϕ^m^ that is the probability that 2 sequences will match for m points without allowing self-counting [62]:



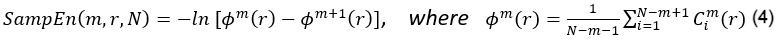



Where *m* denotes the embedding dimension, tolerance r equals 0.1∗*SD*, N denotes the number of data points, and *C_i_^m^* counts, within the tolerance resolution r, the number of matching blocks across different embedding dimensions.

SampEn is a tool used to analyze physiological time-series data, but it does not evaluate the complexity of the data at different time scales. Hence, we applied multiscale entropy (MSE) analysis on raw PPG signals to evaluate the hypothetical difference in signal complexity across various time scales for normoglycemia and elevated glucose levels. However, the scale factor was inversely proportionate to the number of data points. From our empirical results, we found that a minimum of 240 pulse waves were required to correctly compute the MSE values over all the timescale factors (τ=20). We found that the sample entropy calculated from PPG signals during periods of elevated blood glucose was significantly higher than that of blood glucose in the normal range at timescale factors between 8 and 14 (τ). This information was then used to create features for the detection of elevated blood glucose levels. Each timescale factor between 8 and 14 was used as a separate feature. In addition, the mean of the adjacent timescale factors was derived to create additional features. These MSE features are represented in the feature vector with feature indices F225 to F244.

## Results

### Software

All experiments and analyses were performed using Python (version 3.9) and relevant libraries (Table 2). The final model was deployed on Amazon Web Services.

**Table 2:** A list of the software, and relevant libraries, along with the versions used.

Library	Version
Python	3.9.10
Imbalanced-learn	0.10.1
Joblib	1.2.0
Jupyter	1.0.0
Lightgbm	3.3.4
Matplotlib	3.6.2
Neurokit2	0.2.2
Nolds	0.5.2
Numba	0.53.1
Numpy	1.23.5
Pandas	1.5.2
Pillow	9.3.0
Scikit-learn	1.1.3
Scipy	1.8.0
Seaborn	0.12.1
Spectrum	0.8.1
Statsmodels	0.13.5
Xgboost	1.7.2

### Feature Selection

Considering AI ethics and the practicality of implementing the algorithm, some demographic data, such as skin color, race, and personal lifestyle habits, were not used as inputs to the models. However, other general personal characteristics associated with the risk of developing T2DM, such as age, gender, BMI, and family health history of diabetes, were added to the feature vector before the feature-selection process.

The redundant or irrelevant features might hinder the performance of the prediction model. To reduce the dimensionality of the input features, we applied an ensemble strategy that uses multiple feature-selection algorithms. This creates an optimal feature subset that minimizes the prediction error rate and is the most relevant for predicting the target variable. The ensemble feature-selection steps are summarized as follows:

- Six feature-selection methods, including ANOVA correlation coefficient, mutual information, dispersion ratio, recursive feature elimination, lasso regression, and Extreme Gradient Boosting, were used to choose the 30 best features independently.
- We combined the features obtained from each feature-selection method and ranked them using a majority vote approach to find the common features selected by more than 1 model.
- The highly correlated features were dropped from the selected feature subset.

In total, 12 features were selected from the entire feature set and ranked based on the results of the feature-selection strategy (Table 3). In our study, these selected features were the most sensitive predictors for capturing the characteristics of a participant’s elevated blood glucose levels.

**Table 3:** The selected top features after the ensemble feature-selection method.

Rank	Feature
1	Welch_hf_rel
2	AR_hf_rel
3	A_FE_mean
4	A_ratio_mean
5	Age
6	A_Pulse_iqr
7	KTE_skew
8	LOG_std
9	BMI
10	MSE_sum_13_14
11	Family history
12	A_ratio_max
13	Gender^a^

^a^Note that gender was not selected as a top feature in our feature-selection algorithm. However, it was previously identified as a sensitive predictor for T2DM, in which the prevalence of T2DM in men was higher than that in women [63]. This discrepancy could be attributed to the gender imbalance in the data set (men: 10.2%; women: 89.8%). Therefore, we included gender as one of the top features to provide a complete user profile for future investigation and development.

The selected features could be further divided into 4 main categories. Under the time-domain features, the selected features were the area under the PPG curves. A_FE_mean refers to the average area under the falling edge of each pulse (Figure 4). A_ratio refers to the ratio of the area under the rising edge to the area under the falling edge of each pulse (Figure 4), and both the average and maximum values were deemed relevant to the model’s predictions. A_pulse_iqr refers to the IQR of the total area under each pulse (Figure 4). In the frequency domain, the selected features were the relative powers of the high-frequency bands in both the Welch power spectral density (PSD; Multimedia Appendix 1, F32-F44) and autoregressive PSD (Multimedia Appendix 1, F45-F57).

In the nonlinear domain, the selected features were either related to the energy or the complexity of the signal. LOG_std refers to the SD of log-energy entropy (equation 3), whereas KTE_skew refers to the skewness of the KTE energy measure for each sliding window (equation 2). Furthermore, the complexity feature that was selected was the sum of the MSE over 2 scales, 13 and 14.

Finally, the remaining selected features were demographic features that described the age and BMI of the participants, as well as if they had any family history of diabetes.

### Machine Learning Model Performance

Seven widely used machine learning (ML) algorithms, including the naive Bayes classifier, K-nearest neighbors algorithm, logistic regression, random forest, SVM, XGB, and light gradient boosting machine, were trained with the selected features as inputs. We fine-tuned the hyperparameters of each model and validated their performance using the stratified 10-fold cross-validation method. We adopted multiple regularization techniques across various models to prevent overfitting during the model training. Six evaluation metrics, accuracy, sensitivity, specificity, precision, geometric mean (G-mean), and *F* score, were used to evaluate the model’s performance, as accuracy alone cannot provide a comprehensive examination of model performance due to data imbalance. The G-mean and *F* score are critical evaluation criteria to assess the models’ performance, as they are robust to significant label imbalance.

The prediction results from each model are reported as the mean and SD of the evaluation metrics, and Table 4 shows the summary of the results. SVM with the radial basis function kernel showed the best prediction performance with an average accuracy of 84.7%, a sensitivity of 81.05%, a specificity of 88.35%, and a precision of 87.51%. In particular, the average G-mean was 84.54% and *F* score was 84.03%.

**Table 4:** The prediction results obtained from 10-fold cross-validation using various machine learning models.

Model	Accuracy			Sensitivity			Specificity			Precision			Geometric mean			*F* score	
	μ	σ	μ		σ	μ		σ	μ		σ	μ		σ	μ		σ
NB^a^	60.51	4.63	66.17		7.44	54.87		5.78	59.43		4.12	60.08		4.6	62.51		5.19
KNN^b^	76.7	3	90.45		4.30	62.94		4.15	70.97		2.47	75.4		3.09	79.5		2.68
LR^c^	63.1	4.65	64.56		7.07	61.66		4.30	62.65		4.16	63		4.67	63.52		5.37
RF^d^	76.76	5.73	76.84		8.18	76.69		6.42	76.81		6.08	76.64		5.72	76.68		6.23
SVM^e^	84.7	4.14	81.05		6.77	88.34		4.19	87.51		4.26	84.54		4.18	84.03		4.58
XGB^f^	78.06	4.91	77		6.58	79.12		4.98	78.7		4.88	78		4.89	77.77		5.15
LGBM^g^	77.9	3.98	75.54		7.36	80.27		4.45	79.35		4.1	77.74		4.07	77.24		4.81

^a^NB: naive Bayes.

^b^KNN: K-nearest neighbors.

^c^LR: logistic regression.

^d^RF: random forest.

^e^SVM: support vector machine.

^f^XGB: Extreme Gradient Boosting.

^g^LGBM: light gradient boosting machine.

### Model Interpretation

The use of deep learning in the medical and health care domain has shown great potential for solving a range of problems, such as detecting specific symptoms or abnormalities [64,65]. However, the interpretability of deep learning models remains a significant challenge, and it is often difficult for clinicians to trust the decisions made using a black-box system. The lack of model interpretability also raises ethical concerns, particularly when the decision fails. Furthermore, our current data set is considerably small (500 participants) compared with typical deep learning models in other domains, which are trained with thousands of data points. Deep learning models are known to perform well with a larger data set and fail to learn meaningful representations when there is a lack of data [66]. Therefore, we did not investigate the use of deep learning in this study.

As the proposed ML model is designed to complement the existing diabetes detection solution and is relatively new to the clinical community, the features selected in the previous section must be interpretable and exhibit a certain level of agreement with existing findings. A family history of diabetes, being male, being aged ≥45 years, and having an increased BMI have been identified as major risk factors in the literature for developing prediabetes or T2DM [63,67,68]. These 4 risk factors were part of the selected predictors, and this paper provides a preliminary attempt to explain how the selected predictors contribute to detecting elevated blood glucose using the Shapley additive explanations (SHAP) framework. SHAP is a game theoretical approach that provides global and local explanations of the association between the ML output and input features [69].

**Figure 5:**
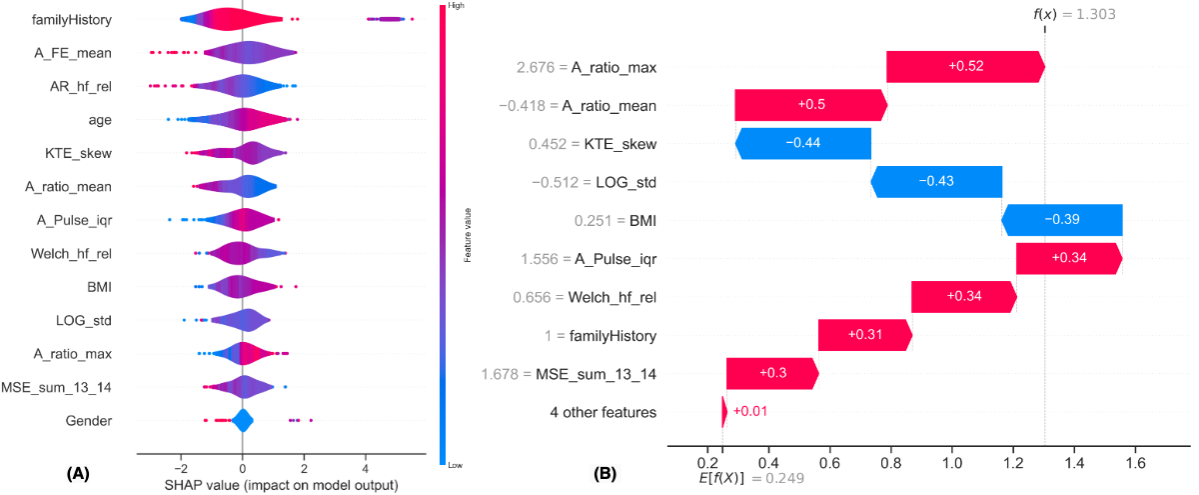
The Shapley additive explanations (SHAP) plots indicate the association between the selected features and their impact on the predicted outcome. (A) SHAP beeswarm plot and (B) SHAP waterfall plot.

Figure 5A illustrates the SHAP values of each feature across all the predictions from the training set. The features were ranked by their mean SHAP values, with larger values shown in red and smaller values shown in blue. The beeswarm plot revealed that a family history of diabetes, increasing age, and higher BMI are associated with a higher probability of elevated blood glucose levels. These observations are consistent with previous research and demonstrate that the ML algorithm has successfully captured the relationship between these features and elevated blood glucose levels. In addition, other proposed features showed varying levels of impact on the model’s output. However, the gender feature did not have any apparent effect on the model’s predictions.

In Figure 5B, each row in the plot shows how the contributions of different features move the output of the model from the expected value (*E*[*f*(*x*)]) to the actual prediction output *f*(*x*) for a single sample with a positive class prediction (blood glucose level ≥7.8 mmol/L) in the test set. The expected value, *E*[*f*(*x*)], is determined using the entire training data set. As expected, most features provide positive SHAP values in this sample, which collectively push the model’s output toward the correct prediction. However, this specific test participant’s BMI was in the healthy range, which pushed the model’s output toward the normal class and might have resulted in a false negative prediction. This indicates that relying on a single feature or demographic data alone may not provide an accurate prediction of blood glucose levels.

Using the SHAP values, we can understand the model’s overall behaviors and how features affect the output positively or negatively, which can help improve the prediction model in the future.

### Assessment of the Elevated Blood Glucose Levels From Multiple Measurements

Generally, diagnostic tests are not highly sensitive and highly specific. Therefore, repeated measurements of the wrist-worn wearable device were combined and assessed in an optimum fashion to maximize sensitivity, specificity, and precision.

Consecutive measures of blood glucose were combined in parallel using the “AND” and “OR” rules to assist in the detection of elevated blood glucose measurement levels. The “OR” rule increases the overall sensitivity, and the “AND” rule increases the overall specificity, which is greater than that of either test alone [70].

## Discussion

### Principal Findings

While the health care landscape is changing, the rapidly aging society and the need for improved population health outcomes call for new models of care to effectively prevent the onset and delay the progression of chronic diseases. Furthermore, short-term health behaviors contribute significantly toward long-term health outcomes, while unattended and frequent glucose spikes might result in prediabetes and eventually diabetes. The availability of noninvasive and device-agnostic blood glucose detection solutions will allow for more frequent and better monitoring of blood glucose levels, thereby reducing the risk of developing T2DM. This study demonstrates that a noninvasive method of assessing diabetes risk using PPG is a viable option to provide a cheaper and accessible modality for the population-wide screening of blood glucose levels. This population-based screening would allow for the earlier detection of DM in the population, especially among those individuals who are unaware of their elevated blood glucose levels. Hence, timely and appropriate lifestyle advice and medical interventions can be provided to prevent diabetes complications. This will subsequently reduce the health care burden for both the individual and the society.

BGEM is a cloud-based solution that can frequently monitor multiple digital biomarkers with minimal disruption to daily life. Developed using the advanced ML operations practice, BGEM can be easily scaled to meet the increasing demand for health care services. The solution includes a user-friendly mobile app that can screen a large population to identify high-risk individuals, people with undiagnosed diabetes, and those who require primary prevention intervention. It also provides timely feedback to users through the app, informing them of their diabetes risk and providing targeted, actionable insights to empower them to take a proactive approach to monitor their glucose levels.

### Limitations

Our pilot study has certain limitations. Since fasting blood glucose measurements were excluded and the criteria to define normal and abnormal levels under fasting conditions differed from our current cut-off, we must refrain from definitively concluding that our model is applicable to fasting conditions. Regarding gender, our feature-selection model did not specifically incorporate it, and our analysis using SHAP demonstrated that gender exerted minimal influence on model predictions. Moreover, all analyses were adjusted for the covariate gender, as required. Therefore, we considered gender to have a limited impact and is not a primary limitation of our findings. To address these limitations, we are actively planning the subsequent phase of data collection. This phase will involve collecting fasting blood glucose measurements in a primary care setting, also allowing for a more balanced gender distribution. More importantly, we could expand our participant pool to encompass participants with prediabetes and diabetes. By addressing these gaps, we aimed to offer a more comprehensive and robust assessment of our model’s applicability and effectiveness.

There was no longitudinal follow-up of the participants. External validation of our model on an independent sample must be undertaken to further assess the detection accuracy and generalizability of the results. Nevertheless, as a preliminary investigation, the potential implications of our findings are significant as they might offer a means to identify previously undiagnosed prediabetes or diabetes cases at the population level. We anticipate that our study will serve as a foundational stepping stone, paving the way for more comprehensive diabetes research using AI and wearable devices. To the best of our knowledge, there is no publicly available data set that systematically examines the relationship between PPG data and blood glucose levels. Acquiring a substantial volume of data is imperative, encompassing a diverse and representative sample spanning the entire spectrum of glucose values and incorporating relevant sociodemographic factors. Such comprehensive data can be obtained through a collaborative effort involving research institutions and industry partners while ensuring strict adherence to local ethical considerations and data privacy regulations.

We demonstrated that the cloud-based ML model can detect elevated blood glucose levels, where consecutive measurements can be combined in an optimal manner to provide high sensitivity, specificity, and precision. However, further research is required to address these limitations.

### Conclusions

In this study, we performed sophisticated feature engineering and found that the features derived from the MSE analysis of PPG signals effectively detect blood glucose changes. We will discuss this set of novel features in detail in a separate paper. To reduce bias and evaluate the generalizability of the model, we used a 10-fold cross-validation to assess its performance. The SVM with the radial basis function model performed the best, with an average accuracy of 84.7%, a G-mean of 84.54%, and an *F* score of 84.03%. Previous models were developed using smaller samples and have lower model performance measures. Our model was developed with a larger sample of 500 participants, and most participants were assessed before and after the consumption of a sugary drink. It also achieved better detection accuracy.

## Appendix

Multimedia Appendix 1 Features summary.

## Abbreviations

AI: artificial intelligence
BGEM: blood glucose evaluation and monitoring
CWT: continuous wavelet transform
DM: diabetes mellitus
G-mean: geometric mean
HR: heart rate
HRV: heart rate variability
IFG: impaired fasting glycemia
IGT: impaired glucose tolerance
KTE: Kaiser-Teager energy
ML: machine learning
MSE: multiscale entropy
PNS: parasympathetic nervous system
PPG: photoplethysmography
PSD: power spectral density
SampEn: sample entropy
SHAP: Shapley additive explanations
SNS: sympathetic nervous system
SVM: support vector machine
T1DM: type 1 diabetes mellitus
T2DM: type 2 diabetes mellitus
