# Supplementary material for: Assessing Elevated Blood Glucose Levels Through Blood Glucose Evaluation and Monitoring Using Machine Learning and Wearable Photoplethysmography Sensors: Algorithm Development and Validation
Source: JMIR AI. 2023 Oct 27;2:e48340. doi: 10.2196/48340 (PMC11041426; doi:10.2196/48340)
Supplement: Multimedia Appendix 1 [file ai_v2i1e48340_app1.docx]

| Index | | Feature | Description and equation |
| --- | --- | --- | --- |
| **HRV^a^ time-domain features** | | | |
|  | F1 | SDNN | The SD^b^ of PPIs^c^ |
|  | F2 | SDSD | The SD of the successive difference between adjacent PPIs |
|  | F3 | RMSSD | Root mean square of successive HRV |
|  | F4 | pNN20 | Percentage of successive PPIs that differ more than 20 ms |
|  | F5 | pNN50 | Percentage of successive PPIs that differ more than 50 ms |
|  | F6 | BPM | Beats per minute |
|  | F7-15 | PPI^c^ | Time difference between two consecutive systolic peaks |
|  | F16-F24 | HRV^d^ | Time difference between 2 consecutive PPIs |
| **HRV frequency-domain features** | | | |
|  | F25-F31 | AR^e^ coefficients | AR coefficients were used to represent the change in the shape of the pulse occurring due to a change in blood flow. To ensure that AR model accurately captures the shape of the pulse, we used AR model of order 7 and compute the coefficients using the Yule-Walker equation, which is derived from sample covariance:  $\sum_{K=1}^{N} a_{k}\gamma_{xx}\left[ l-k \right]=-\gamma_{xx}\left[ l \right]$ |
|  | F32-F44 | Welch power | Absolute, relative, log, and normalized power of the VLF^f^, LF^g^, and HF^h^ bands computed using Welch method. The total power across all frequency bands was also computed. |
|  | F45-F57 | AR power | Absolute, relative, log, and normalized power of the VLF, LF, and HF bands computed using AR method. The total power across all frequency bands was also computed. |
|  | F58-F60 | Welch peak | The peak frequency of the VLF, LF, and HF bands were computed using the Welch method |
|  | F61-F63 | AR peak | The peak frequency of the VLF, LF, and HF bands were computed using the AR model. |
|  | F64-F65 | LF/HF | The ratio of LF to HF power was computed using the AR and Welch method. |
| **HRV nonlinear domain features** | | | |
|  | F66 | Area | Area of the ellipse that represents total HRV |
|  | F67 | SD1 | Poincaré plot SD perpendicular to the line of identity |
|  | F68 | SD2 | Poincaré plot SD along the line of identity |
|  | F69 | SD1/SD2 | Ratio of SD1 to SD2 |
|  | F70 | DFA^i^ α1 | DFA, which describes short-term fluctuations |
|  | F71 | DFA α2 | DFA, which describes long-term fluctuations |
| **Heart rate features** | | | |
|  | F72-F81 | HR statistics^d^ | Heart rate statistics |
| **Continuous wavelet transform features** | | | |
|  | F82-F84 | CWT^j^ | The CWT was performed on the PPG^k^ signal using the Mexican Hat Wavelet. The mean, SD, and maximum value of the resulting CWT values were used. |
| **Waveform features** | | | |
|  | F85-94 | RP^d^ | Magnitude of rising edge peak for PPG signal |
|  | F95-104 | FN^d^ | Magnitude of falling edge notch for PPG signal |
|  | F105-F114 | RT^d^ | Rising time *RT*=t*_p_*−t*_s_* (t*_s_*: start time of the current waveform; t*_p_*: the peak time of the current waveform) |
|  | F115-F124 | FT^d^ | Falling time *FT*=t*_e_*−t*_p_* (t*_e_*: end time of the current waveform) |
|  | F125-F134 | AUR^d,l^ | Area under rising edge |
|  | F135-F144 | AUF^d,m^ | Area under falling edge |
|  | F145-F154 | Apulse^d,n^ | Area under one pulse or waveform Apulse=*AUR+AUF* |
|  | F155-F164 | Aratio^d^ | Aratio=$\frac{AUR}{AUF}$ |
|  | F165-F174 | Rslope^d^ | Slope of rising edge Rslope=$\frac{f\left( t_{p} \right)-f\left( t_{s} \right)}{t_{p}-t_{s}}$ |
|  | F175-F184 | Fslope^d^ | Slope of falling edge Fslope=$\frac{f\left( t_{e} \right)-f\left( t_{p} \right)}{t_{e}-t_{p}}$ |
|  | F185-F194 | Timediff^d^ | Timediff=$RT-FT$ |
|  | F195-F196 | Eigenvalue | The first and second eigenvalue of the first derivative of PPG signal |
| **Energy features** | | | |
|  | F197-F206 | KTE^d,o^ | Kaiser-Teager energy |
|  | F207-F223 | LogE^d^ | On top of the statistical parameters of log energy (LogEn), *LogE_n_*, was used to compute AR coefficients of order 7 |
| **Complexity measures** | | | |
|  | F224 | SampEn | Sample entropy |
|  | F225-F244 | MSE^p^ | Multiscale entropy reveals the confidence of entropy measures on the scale by quantifying the time series’ complexity |
| **Patient demographics** | | | |
|  | F245 | Age | Age of the participant |
|  | F246 | BMI | BMI |
|  | F247 | Family history | If an ancestor had diabetes |
|  | F248 | Gender | Gender of the participant |

^a^HRV: heart rate variability.

^b^SD: standard deviation.

^c^PPIs: peak-to-peak intervals.

^d^Statistical parameters, such as mean, median, SD, skewness, kurtosis, minimum, maximum, IQR, mean absolute difference, and the difference between the mean and median were computed.

^e^AR: autoregressive.

^f^VLF: very low frequency.

^g^LF: low frequency.

^h^HF: high frequency.

^i^DFA: detrended fluctuation analysis.

^j^CWT: continuous wavelet transform.

^k^PPG: photoplethysmography.

^l^AUR: area under the rising edge.

^m^AUF: area under the falling edge.

^n^Apulse: area under a PPG wave.

^o^KTE: Kaiser-Teager energy.

^p^MSE: multiscale entropy.
